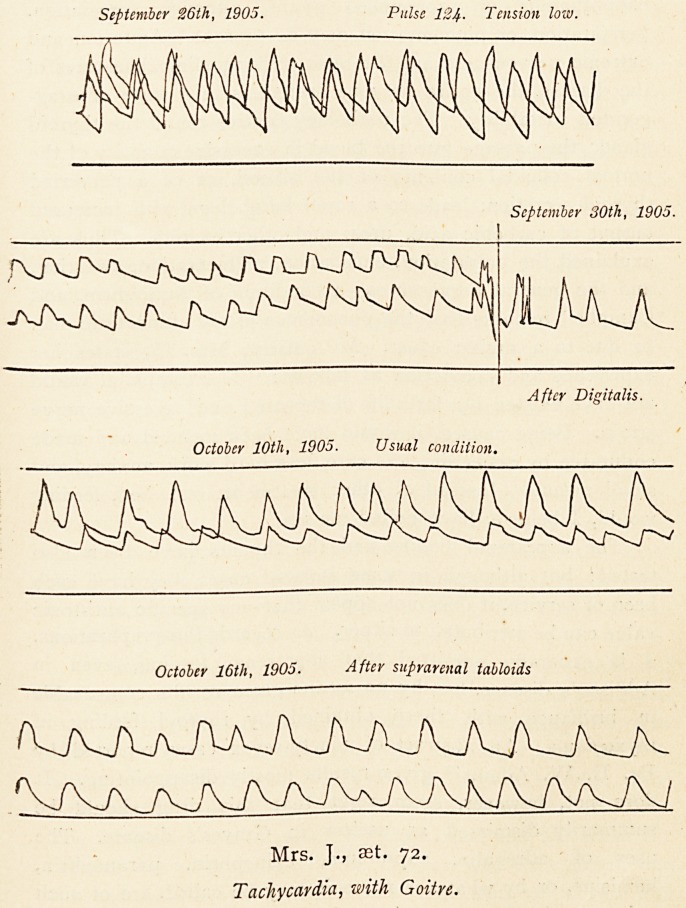# The Long Fox Lecture

**Published:** 1905-12

**Authors:** R. Shingleton Smith

**Affiliations:** Emeritus Professor of Medicine, University College, Bristol; Consulting Physician to the Bristol Royal Infirmary; and Honorary Fellow of King's College, London


*Thyroid.—Normal.*

*Thyroid.—Graves's Disease.*

### THE LONG FOX LECTURE:

THE SECOND ANNUAL LECTURE ARRANGED BY THE COMMITTEE OF THE LONG FOX MEMORIAL,

DELIVERED IN THE MEDICAL LIBRARY, UNIVERSITY COLLEGE, BRISTOL, ON DECEMBER 7TH, 1905.

NELSON C. DOBSON, F.R.C.S., *in the Chair.*

BY

R. SHINGLETON SMITH, M.D., B.Sc. Lond.,

*Emeritus Professor of Medicine, University College, Bristol; Consulting Physician to the Bristol Royal Infirmary; and Honorary Fellow of King's College, London.*

ON

### THE PATHOLOGY AND TREATMENT OF GRAVES'S DISEASE.

---

"All the world 's a stage,
And all the men and women merely players."

"Ring down the curtain, for the play is done;
Let the brief lights die out, and darkness fall,
Yonder to that real life he has his call:
And the loved face beholds the Eternal Sun."

"*Punch*" *on Sir Henry Irving*, Oct. 18th, 1905.

---

EDWARD LONG FOX's seventy years of life and work having ended on March 28th, 1902, it was very shortly afterwards determined by his numerous friends that steps should be taken to establish an annual lecture on some subject connected with medical science, to be delivered at the University College, Bristol, and to be known as the Long Fox Lecture. Accordingly in 1904 the first of the series was given by Fox's life-long friend, the venerable physician and anthropologist, Dr. John Beddoe, F.R.S., and his subject was, "The Ideal Physician."

Oliver Wendell Holmes, in *Elsie Venner*, made the suggestion that "it is by no means certain that our individual personality is the single inhabitant of these our corporeal frames. . . . . Some at least, who have been dead, may enjoy a kind of secondary and imperfect, yet self-conscious life, in these bodily tenements which we are in the habit of considering exclusively our own. . . . . This body in which we cross the isthmus between the two oceans is not a private carriage, but an omnibus."

If this be so, and our old friend Fox was able to be more or less present with us on that occasion, how gratified he would have been with the oration which was then delivered, what a pleasure it must have been to see his old colleague's youthful fire warm up to the occasion, whilst he described in eloquent language the ideal physicians of the past, and more particularly the one who had recently departed. This, the inaugural lecture, "of a series instituted to honour and commemorate an able, a laborious, an unselfish and generous physician and philanthropist, a man who loved his profession and his fellow creatures," was on a subject of general interest to a non-professional as well as a medical audience; but it has been thought desirable that on the second occasion the lecturer should select some concrete medical topic, giving an account of the recent advances of science in some department of medicine or surgery in which he may be especially interested, and which will be appropriate to a professional rather than a lay audience.

In endeavouring to carry out this view I must first express a word of profound regret, which will be shared in by all present, that the untimely bereavement of the newly-elected Emeritus Professor of Medicine in this College, Dr. Markham Skerritt, so many years Dean of the Medical Faculty, should have given rise to the (I hope only temporary) relinquishment of the duty which he had undertaken to lecture to you to-day.

In accepting the invitation of the Committee to take up this duty I felt that my audience would not be severely critical, but would be indulgent to any shortcomings of my own which may partially arise from the brief period left to me in which to make preparations for and with which to illustrate the Long Fox Lecture of 1905.

My choice of a subject was not determined without some hesitation. After reflecting for some days, it occurred to me that some of Long Fox's own work would be an appropriate topic. The Bradshaw Lecture of 1882 was "On the Influence of the Sympathetic on Disease," and this also was the title of the principal volume[1] which Fox published three years later. Much new work has since been added to that which he wrote; but I propose to limit myself to one chapter only of his work, that on the pathology and therapeutics of Graves's disease. A consideration of this interesting malady gives me an opportunity of directing attention to some of the work of Edward Long Fox, also to some investigations of my own carried on a few years ago, and further to many perhaps more interesting and possibly useful developments which centre round the thyroid gland in relation to the circulation and the heart. I shall therefore have to inquire what were the views in vogue when Long Fox wrote on this subject, and how have they been modified since that time.

In Chapter V. of Fox's book we see what was the most advanced view of the disease even so recently as twenty years ago. After quoting numerous cases in which certain coarse lesions in the cervical sympathetic had been found in association with this group of symptoms, amongst them a case of my own in which there was marked shrinking of the cells of the right inferior cervical ganglion with the conversion of the left ganglion into a calcareous nodule, Fox admitted that "it is not possible to credit this system of nerves with the causation of all the symptoms," and he came to the conclusion that "whilst the central origin theory gives the only reasonable explanation of the palpitation and of Graefe's phenomenon, it accounts for the other important symptoms of exophthalmic goitre by the lesion being of a vaso-motor centre, the effects being modified and specially localised by the influence of the cervical sympathetic."

The varying views as to the pathology of the condition were then summed up by Fox as follows:—

"Basedow says the disease is the result of a scrofulous dyscrasia; Aren calls it a neurosis of the sympathetic; Trousseau, a congestive neurosis of the whole ganglionic system; Charcot believes in a psychical origin; Jaccoud considers it a paralysis of the vaso-motor centres and cervical ganglia, and says that the beating of the heart is due to the less resistance in the dilated vessels, that this dilatation is increased by the heart-beats, that thence there is tumefaction of the thyroid gland, that the cerebro-spinal axis is in its turn excited by the peculiar blood-flow, that the brain shows psychical disturbance, and the cilio-spinal region produces exophthalmos by spasm of the orbital muscles and of the muscles of Müller, and dilatation of the pupil by spasm of the radiating fibres of the iris; Daviller believes it to be an anæmia of the cilio-spinal regions of the cord causing an exaggeration of the reflex power; whilst Eulenberg and Guttmann think it a paralytic condition of the cervical sympathetic."

It does not seem to be possible to dismiss the nervous system altogether as of no importance in the pathological questions which centre round Graves's disease, for even Dr. Greenfield,[2] in 1893, described many changes found in the nervous system, and as regards the sympathetic in the neck he states that "very marked changes have been found. . . . From a comparison of these changes there can be no doubt that the sympathetic ganglia are the seat of a sub-acute inflammatory process. It may be fairly assumed that this leads to changes in function, either by irritation or degeneration. The contrast of similar parts in myxœdema is very striking." As regards the central nervous system, he remarked that "the changes seen resembled in general character those seen in tetanus and hydrophobia, less in degree." Many other observers concur in this view that although the changes in the peripheral ganglia and in the higher centres may not be primal and final, yet they are real, and are indicative of a grave toxæmic condition allied to the changes found in many other toxic states. We cannot even yet look upon the thyroid gland as everything and the whole physiological world around it as nothing, neither can we consider the nerve centres to be everything and everything else as nothing.

The long-accepted discovery first demonstrated by Brown-Séquard, and afterwards confirmed by Filehne and others, that the cardinal symptoms of Graves's disease may be induced in dogs and in rabbits by wounding and irritating the restiform bodies, have recently derived some support from observations on the higher centres of the nervous system made by Tedeschi,[3] who contends that the disease may be induced in dogs by lesions of the anterior portion of the restiform bodies. He claims that the disease is due to some anatomic or functional lesion of these bodies, probably connected with the vagus or sympathetic or with the vaso-motor centre. This bulbar alteration is followed first by hyperæmia, then hyper-secretion of the thyroid gland, and this in turn produces other symptoms, including the changes of metabolism.

It cannot fail to be noted that so recently as 1885 there is no mention of any serum, vaccine, toxin, or anti-toxin; at that time it had not occurred to anyone that the widely-spread symptoms of this striking disease could be due to an auto-intoxication caused by the thyroid gland, and that the condition would in course of time come to be looked upon as the antithesis of myxœdema.

My share in this work has been mainly of a negative character. The case mentioned by Long Fox[4] was that of a girl, twenty years of age, with severe symptoms of seven months' duration, whose persistent tachycardia (pulse being as high as 180 to 208), cough, dyspnœa, and cyanosis culminated in heart failure. After the autopsy the hypertrophic thyroidal changes were described in detail, and also various pathological conditions of the sympathetic nerves and cervical ganglia. For some years after this time I systematically examined the cervical and the semilunar ganglia taken from the bodies of patients dying of many diseases, and came at last to the conclusion that the mode of preparation of the specimens was of more importance than the cause of death of the patient, and that the changes visible in the microscopic character of the cells of the ganglia had little connection with the symptoms present during life. Accordingly it has gradually become the custom to look to some other cause than either the central nervous system or the peripheral sympathetic and vaso-motor nerves for an explanation of the phenomena of Graves's disease.

The President of the Bristol Medico-Chirurgical Society, in his recent address (page 305), spoke of the collection of living pictures, we might call them cerebrographs, gathered by the practitioner in a long course of years. . . . He spoke of them as "a huge reference library that we, and we alone, can utilise for the benefit of our clients. . . . With these pictures stored away in the pigeon-holes of our memory, it is far easier to call upon them for reference than to refer to the laborious and arid notes that lie hidden away at home in a book that is possibly unindexed."

Oliver Wendell Holmes works up the same idea when he makes Dr. Kittredge observe:—

"When a man that 's once started right lives among sick-folks for five and thirty years, as I 've done, if he has not got a library of five and thirty volumes bound up in his head at the end of that time he 'd better stop driving round and sell his horse and sulky. I know the bigger part of the families within a dozen miles' ride. I know the families that have a way of living through everything, and I know the other set that have the trick of dying without any kind of reason for it. I know the years when the fevers are in earnest, and when they are only making believe. I know the folks that think they are dying as soon as they are sick, and the folks that never find out that they are sick until they are dead. You can't tell a horse by driving him once, nor a patient by talking half an hour with him."

Long Fox was such a clinical observer, and he had many volumes of mental pictures with which to instruct. From his training and matured experience we may well say of him in W. E. Henley's words, that he was able—

> "To speak Latin with a right accentuation,
> And give at need (as one who understood)
> Draught, Counsel, Diagnosis, Exhortation."

It might also be said of him, as of Lord Lister—

> "His wise, rare smile is sweet with certainties,
> And seems in all his patients to compel
> Such love and faith as failure cannot quell."

But clinical work and experimental investigation are not always compatible ; we can all realise how difficult it is to carry on any experimental investigation in the routine, every-day life of the physician in active work. Kipling has given us a word-picture of this when he wrote something as follows :—

> "When through the gates of stress and strain
> Comes forth the vast event,
> The simple, sheer, sufficing, sane
> Result of labour spent.
> They that have wrought the end well-thought
> Be neither saint nor sage,
> But men who merely did the work
> For which they drew the wage."

Clinical research with most of us has to be carried on under difficulties of recording and remembering which increase as life advances. Fox often did the well-thought work regardless of the wage, just as the illustrious Jenner of our own county was able in his country work to make an undying name by "proving all things," and I remember that our anthropological lecturer of last year, when doing clinical work, always found time to note the colour of the eyes and hair, often also the diameters of the head.

Since Fox's time the research work of the new era of bacteriology has tended to obscure the clinical work of the physician ; but, nevertheless, clinical observation is as necessary as ever, and there are other causes of disease than toxins and other remedies than anti-toxins. Considerations to which I have alluded must be my plea for any incompleteness to-day of my story of the pathology and treatment of Graves's disease. I have not kept an accurate record of all cases, and the mental pictures often fail in completeness and accuracy. I cannot, therefore, give you an analysis of the details of some hundreds of cases, such as that given recently by Dr. George Murray in his Bradshaw Lecture of 1905. My facts must be mainly those gleaned by others, and it is interesting to note how many of these special lectureships have to be quoted in connection with the subject which has attracted so much attention during the last few years.

Now it is necessary to go back a century earlier than the date of Long Fox's book, for it was in 1786 that an English physician, Caleb Hillier Parry, commented on the association of the thyroid gland with the heart, when he wrote as follows:—

"There is one malady which I have in five cases seen coincident with what appeared to be enlargement of the heart, and which, so far as I know, has not been noticed in that connection by medical writers. The malady to which I allude is enlargement of the thyroid gland."

The next distinct recognition of the cardio-thyroid condition was that of Robert J. Graves, who in one of his clinical lectures that on functional disease of the heart, first published in 1835, commented on the connection between palpitation of the heart and enlarged thyroid. He said,[5] and it is of interest to quote the exact words of one who was described by Trousseau as a perfect clinical teacher: "I have lately seen three cases of violent and long-continued palpitations in females, in each of which the same peculiarity presented itself, viz. enlargement of the thyroid gland: the size of this gland, at all times considerably greater than natural, was subject to remarkable variations in every one of those patients. When the palpitations were violent, the gland used notably to swell and become distended, having all the appearance of being increased in size, in consequence of an interstitial and sudden effusion of fluid into its substance. The swelling immediately began to subside as the violence of the paroxysm of palpitation decreased, and during the intervals the size of the gland remained stationary. . . . The sudden manner in which the thyroid, in the above three females, used to increase and again diminish in size, and the connection of this with the state of the heart's action are circumstances which may be considered as indicating that the thyroid is slightly analogous in structure to the tissues properly called erectile. . . . The enlargement of the thyroid, of which I am now speaking, seems to be essentially different from goitre in not attaining a size at all equal to that observed in the latter disease. . . . The well-known connection which exists between the uterine functions of the female and the development of the thyroid observed at puberty renders this affection worthy of attention, particularly when we find it so closely related by sympathy to those palpitations of the heart which are of so frequent occurrence in hysterical and nervous females."

In later years Gull's description of cretinism in the adult in 1873, followed by Ord's description five years later of the atrophic condition of the thyroid in the disease which he named myxœdema, the demonstration of the results of extirpation of the thyroid, then known as Kocher's cachexia strumipriva, and finally Horsley's investigations, were all leading up to the conclusion stated by Moebius in 1886, that an over-functioning thyroid causes exophthalmic goitre, just as a non-functioning gland produces myxœdema, whilst it was in 1896 that Horsley[6] declared that "exophthalmic goitre in its various stages results from perversion of the function of the thyroid gland."

Time would fail me to take up the details of the clinical picture of the disease, accordingly my remarks must be limited chiefly to the pathological aspects of the subject.

In my own case, to which Fox alludes, there was a coarse lesion of the sympathetic chain in the neck, the left inferior ganglion had disappeared, and was replaced by a nodular mass, adhering to the left side of the trachea, of an oval shape, having a dense fibrous capsule into which the nerve fibres could be traced, and containing internally a hard, calcareous, calculus-like mass forming the nucleus of the nodule; this mass dissolved with dilute acid, but a few crystals of cholesterine were seen amongst the undissolved debris. In addition to this defect further microscopic examination of the other ganglia on both sides showed that the nerve cells had undergone striking and characteristic changes. (Figs. 1 and 2.)

The true nucleated and nucleolated cells were visible as granular masses, for the most part stellate in form, surrounded by a clear space, outside which a distinct nucleated cell capsule could be traced. The cells varied much in size; some of them filled about two-thirds the area of the space enclosed by the capsule, others had retracted to such an extent that they appeared to occupy not one-tenth of their normal space; some still remained in direct contact with the surrounding cell sheath, others had broken away entirely and

× 300.

Fig. I.

*Sup. Cervical Ganglion, left.*

*Drawn by* J. Greig Smith.

× 360.

Fig. 2.

*Sup. Cervical Ganglion, right.*

*Drawn by* G. Munro Smith.

appeared to be free within the capsule. Changes of this kind were later confirmed by Prof. Greenfield.

The condition of the thyroid in this case was carefully noted. The gland presented a condition of vascular and villous hypertrophy. The circular spaces which exist normally were not enlarged, no cysts were visible, and there was no colloidal degeneration of the epithelial lining. The spaces were occupied by villous growths, the result of vascular and

× 50.

Fig. 3.

*Section of Thyroid—Graves's disease.*

epithelial proliferation of the walls of the loculi. The villous projections were covered, and the locular spaces were lined by a single layer of epithelium of the flattened columnar variety. (Fig. 3.)

These two conditions, the changes in the sympathetic system and those of the thyroid gland, were the principal morbid appearances in this severe and rapidly fatal case. The changes in the thyroid were at that time thought to be of no importance, and those of the sympathetic were unduly magnified.

A few years later a very remarkable case was reported in detail by Dr. J. Michell Clarke,[7] who also especially commented on the pathological condition of the thyroid gland. He described the vessels in excess and the large amount of epithelial elements in relation to the connective tissue. Besides the acini of the regular round or oval form there were large numbers of irregularly-shaped vesicles. Evidences of rapid proliferation and decay of cells were everywhere present, and in some places the cells reverted to the columnar instead of the glandular spheroidal type. This case was fatal in six weeks from its commencement, death taking place from rapid wasting, vomiting, and diarrhœa. A persistent thymus was found, but although no defects were observed in the nerve centres or sympathetic ganglia, Dr. Clarke endeavoured to show that the cause of the disease must be sought for in the medulla oblongata, the cardio-inhibitory, the diabetic, and the vaso-motor centres being involved. He made no attempt to show that the pathological thyroid gland was of any importance in the etiology of the disease.

It was accordingly reserved for Sir Victor Horsley in his Brown Lectures of 1886 to give the thyroid its full recognition as the most important factor in the causation of cardio-thyroid exophthalmos, and Professor Greenfield in his Bradshaw Lectures of 1893 suggested that the various changes demonstrated (destruction of colloid, cell proliferation, adenomatous, cystic, and finally fibroid degeneration) were truly stages in an alteration, beginning in catarrh and ending in fibrosis or cystic degeneration, comparable to the same range of changes seen in the ovary or the lung.

From this time onwards the nervous system has received correspondingly little attention, and the thyroid gland has been the centre of interest in connection with exophthalmic goitre. It is somewhat remarkable that in spite of the fact that the thyroid was obviously the organ which showed most pathological change, yet this gland was considered to be of so little consequence, either physiologically or pathologically, that its enlargement was considered to be incidental and of practically no importance.

The general structure of the thyroid gland is well known.

Fig. 4.

*Normal Thyroid of Dog.*

Fig. 5.

*Thyroid of Puppy.*

Its closed vesicles, lined with epithelial cells, which secrete the colloid, are the essential parts. The normal vesicles are commonly rounded in shape or angular from compression, and the secreting cells are cubical.

The most striking change in the enlargement of goitre is that the vesicles become branched and stellate, and that the cells become columnar.

Fig. 6.

*Parathyroid, Monkey.*

The parathyroids are intimately associated with the thyroids. They are small glands, formerly passed by as of no account, but they have derived importance from the fact that their excision generally causes the death of the animal. Their structure is quite different from that of the thyroid; they consist wholly of cells, and contain no vesicles or colloid. Dr. Welsh, of Edinburgh, finds that there are four of them—one anterior and inferior to, one posterior and superior to each thyroid lobe.

Many experiments on excision of the thyroid were made by Sir Astley Cooper, Sir John Simon, Schiff, and others, but Victor Horsley's work, carried on during the years 1884 to 1886, fully described in his Brown Lectures,[8] on the pathology of thyroid gland, is the foundation of our recent knowledge.

Having first alluded to the hæmatopoietic function, as shown by the excess of the red corpuscles in the efferent vein, and to the well-known fact that the internal secretion of the thyroid (the colloid matter) passed directly from the acini of the gland into the lymphatics, he then directed attention to the work of Schiff, who in 1884 definitely proved that the removal of the gland was followed by striking symptoms preceding a fatal termination. There was as yet no proof that the loss of the gland was the cause of cretinism or myxœdema, but at this time Horsley, by experiments on monkeys, established beyond question the fact that the typical symptoms of myxœdema could be produced by simple removal of the thyroid, and were due to the loss of the gland itself, and not to injury of its nerves. He considered it to be difficult to understand how the sympathetic theory could have been maintained, and believed that its final destruction had been given by the discovery of Dr. George Murray that the phenomena of myxœdema can be dissipated by the internal administration of the gland itself. He further described and commented on the compensating hypertrophy of one lobe of the gland when the other had been removed, and he described the changes in the epithelium of the enlarged gland.

In exophthalmic goitre the acini become more irregular and more resemble a racemose gland, the colloid material tends to disappear, and is represented by a granular debris. This is coupled with a general enlargement of the whole gland, just as in the artificial hypertrophic compensation. . . . Further, as regards the changes in the epithelium, a great deal has been said by many observers as to the vacuolation of the epithelium and the appearances of vacuolation in the colloidal substance.

To summarise our present position and knowledge of the whole question, said Mr. Horsley in 1896, "It is, I think, now generally agreed that, whereas myxœdema and cretinism result from simple loss of the function of the thyroid gland, exophthalmic goitre in its various degrees results from a perversion of that function."[9]

Walter Edmunds,[10] working at the Brown Institution at about the same time, also demonstrated that the removal of a portion of the thyroid from an animal was followed by a compensating hypertrophy in the remaining parts of the gland. In this hypertrophied gland he found that the vesicles had enlarged, becoming long and branched, that the lining membrane became convoluted, the secreting cells columnar, and the colloid contents of the alveoli were less viscid and more watery. These changes,

Fig. 7.

*Compensatory Hypertrophy of Thyroid after partial Thyroidectomy.*

Fig. 8.

*Thyroid.—Graves's disease.*

artificially produced, he found to be identical with those found in the enlarged thyroid of Graves's disease, and accordingly he inferred that this hypertrophy of the gland is of the nature of a compensating hypertrophy, that it is not primarily of central nerve origin, nor was it in any way affected by division of the sympathetic. He believed the over-growth was due to an unsuccessful attempt at compensation, and therefore it might be inferred that the thyroid changes are secondary to something else, and could not be looked upon as the starting-point of the disease in exophthalmic goitre.

Dr. G. R. Murray,[11] working on similar lines, also demonstrated that the compensating hypertrophy occurring in Graves's disease was not simply due to over-vascularity and hyperplasia of the original glandular tissue, but that considerable changes in structure might be observed. He also found that the alveoli became irregular in outline, with a folding-in of the lining membrane giving projections into the lumen of the alveoli, that the cells increased in size and number, and the form changed from the short cubical to the long columnar epithelium. The colloid contents were diminished and watery in consistence. He summed up his views by stating that "the glandular tissue was working at high pressure, and was just able to supply the necessary amount of secretion without storing any in reserve in the alveoli, as is usual in the normal gland." He found the gland lesions to be more constant than any in the nervous system, and the same enlargement of the gland had been present in almost every one of the cases he had observed. He had found that treatment of the simple parenchymatous goitre by thyroid extract leads to a notable diminution in the size of the gland, but in his opinion thyroid extract should never be given in exophthalmic goitre, as it is only adding fuel to the fire.

An investigation by Hutchison[12] on the active constituent of the thyroid gland led to the conclusion that the total therapeutic activity of the gland is to be attributed to the colloid matter. "In giving the colloid one is giving the active part of the glands, nothing more and nothing less." His preparation is called thyro-colloid.

Then followed numerous observations on the removal of the parathyroid glands from animals; but as regards the human subject little is yet known, and it seems doubtful whether these glands take any part in the phenomena of Graves's disease. The usual result of the removal of the parathyroid glands has been death by tetany, but it has been discovered that this tetany may be counteracted by the injection of parathyroid emulsion in dethyroidised animals. The result of destruction of the thyroids being myxœdema, and that of the parathyroids alone being an acute fatal nerve disease resembling tetany, it follows that the effects of these two sets of glands must if possible be differentiated. The great importance of the parathyroid glands in connection with the experiments on thyroid extirpation was shown by the fact that the preservation of one parathyroid will suffice to preserve the life of a dog from which both thyroids have been removed, and Edmunds found that a compensating hypertrophy of the retained parathyroid occurs.[13] It has been pointed out by MacCallum and Davidson[14] that a comparison of the symptoms following extirpation of the parathyroids shows that although there is a close resemblance to exophthalmic goitre the tachycardia is not present, although exophthalmia may be, and the violent convulsive movements of the hypo-parathyroidal condition are the representatives of the nervous tremor of Graves. It has been thought that the parathyroid feeding may be effective clinically where the thyroid medication has failed; accordingly Gley[15] treated exophthalmic goitre with the parathyroids of the ox, and he reports that Moussu had in one case obtained marked improvement therefrom. In another case twelve raw parathyroids of the ox had been given daily, but with no appreciable effect. More recently Walsh,[16] after some experience of this method, came to the conclusion that there are no grounds for the idea that insufficiency of the parathyroids plays any important part in Graves's disease. A general summary of the parathyroid glands in Graves's disease has just now been given by Lawrence Humphry,[17] who in several fatal cases of exophthalmic goitre found the parathyroid glands to be invaded with extensive infiltration of fat, but in none of them did they show any signs of compensating hypertrophy or tendency to the formation of colloid.

The whole question of the pathology of Graves's disease was recently summed up very tersely by Hector Mackenzie.[18] After commenting on the emaciation which is commonly present in any fatal case, and which is presumably due to the increased metabolism, which is well known to be one of the most striking symptoms of the disease, he points out that the most obvious feature is the thyroid swelling. The gland enlarges, generally in a uniform manner affecting all parts equally, the veins on the surface are dilated, and the nutrient arteries are dilated and tortuous. The substance is soft in consistence excepting in long-standing cases, and on section it is easy to see a marked hypertrophy of the blood vessels. Microscopic examination shows the various changes which have been already described. He further goes on to point out the striking similarity which exists between the microscopic appearances of the gland tissue in Graves's disease and in the case of an animal from which the greater part of the gland has been experimentally removed. He then notes the compensating hypertrophy which occurs in the small portions of the gland remaining after partial extirpation, and that a section of the gland from a case of Graves's disease is practically indistinguishable from one from the gland of an animal with this experimental compensating hypertrophy.

Fig. 9.
*Secreting Cells, Normal Thyroid. Dog.*

Fig. 10.
*Changes in Secreting Cells after partial Thyroidectomy.*

Fig. 11.
*Secreting Cells, Thyroid.—Graves's disease.*

It is therefore shown that in Graves's disease the thyroid, for some reason at present obscure, takes on increased functional activity and undergoes hypertrophy. Further, it seems probable that the parathyroids play some part; atrophy of these has been observed in some cases, but it seems as yet doubtful if this is an essential feature of the disease. The persistence of the thymus gland has been frequently observed, but its microscopic appearance does not differ from that of the normal gland. He concludes his summary as follows: "What it is that starts the hypertrophy of the thyroid and what part the persistent thymus plays we do not know. In some cases the disease seems to be set up by shock or by emotion. . . . It is possible that the disease in some cases may be due to microbic infection."

That the most conspicuous symptoms of Graves's disease are the result of over-activity of the thyroid is shown by the effects which have been obtained by the administration of large doses of thyroid gland in human beings and in the lower animals. Mr. Walter Edmunds has shown that if dogs or monkeys are fed with large doses of thyroid gland there results accelerated action of the heart, increased metabolism, loss of weight, increased action of the skin, and sometimes marked exophthalmos. Indeed, you find that practically all the symptoms of Graves's disease, including exophthalmos, can be produced in animals by the administration of large doses of thyroid gland.

Sajous[19] endeavours to carry the etiology of the disease a stage further back: he admits the effect of thyroid extractives on metabolism, that an increase of physiological activities, such as are seen in exophthalmic goitre in the acute forms, is likely to be due to over-action of the thyroid glands; but he endeavours to show that the thyroids act only as an auxiliary to the adrenal system, that they supply a secretion which stimulates the adrenals, and thereby augments the activity of oxidation processes. He transfers to the adrenals a long list of symptoms commonly thought to be associated with abnormal activity of the thyroid, so that he considers Graves's disease to be the result of excessive supra-renal activity, whilst myxœdema is due to the opposite condition, adrenal insufficiency. Myxœdema has been known to follow exophthalmic goitre,[20] and this he would account for on the principle that excessive thyroidisation has so stimulated the adrenals that this culminates in over-stimulation and exhaustion, giving the result, adrenal insufficiency. Dr. Willcox, of Warminster, tells me that he has under observation, and has for years treated successfully a case of myxœdema, which commenced with exophthalmic goitre. In criticism of these views we must not, however, forget that there are no pathological proofs that the supra-renal capsules are in any way responsible for the phenomena either of myxœdema or of exophthalmic goitre.

The brilliant discovery of Murray that myxœdema could be held in abeyance indefinitely by the administration of thyroid food—the signal triumph over athyrea—has led to great expectations from organo-therapy, and the various therapeutic tests which have now continued for some years are in themselves an important part of the pathological investigation of Graves's disease. Osler remarks[21]: "The use of the extracts of certain organs (or of the organs themselves) in disease is as old as the days of the Romans; but an extraordinary impetus has been given to the subject by the discovery of the curative powers of the extract of the thyroid gland in the diseases known as cretinism and myxœdema." That this fact needs still to be recalled from time to time is well shown by the following story given by Osler (p. 221):—

"It is astonishing with how little reading a doctor can practise medicine, but it is not astonishing how badly he may do it. Not three months ago a physician brought to me his little girl, aged 12. The diagnosis of infantile myxœdema required only a half glance. In placid contentment he had been practising twenty years in 'Sleepy Hollow,' and not even when his own flesh and blood was touched did he rouse from an apathy deep as Rip Van Winkle's sleep. In reply to questions—No, he had never seen anything in the journals about the thyroid gland; he had seen no pictures of cretinism or myxœdema; in fact, his mind was a blank on the whole subject. He had not been a reader, he said, but he was a practical man with very little time."

It is commonly admitted that in simple common goitre thyroid medication is often successful in curing the malady. All forms of goitre begin with abnormal development of the thyroid parenchyma, and no definite line of demarcation exists between simple thyroid swelling and Graves's disease. The question, therefore, naturally arises whether it may be possible that the transition may be promoted by thyroid medication, and hence the necessity for caution in giving thyroid preparations to persons with simple thyroid enlargement without as yet any indications of exophthalmos or tachycardia. A study of the pharmacology of the thyroid gland by Hutchison[22] shows that—

(*a*) It increases metabolism, causing great access of urea, copious loss of fluid and loss of weight, with consumption of the body fat.

(*b*) It causes increased rapidity of heart, with irregularity and palpitation.

(*c*) Its active constituent is excreted by the kidneys, iodine being found in the urine after large doses of thyroid.

(*d*) The dose must be limited, or thyroidism results, giving headache, pains in limbs, nausea, diarrhœa, and palpitation.

It is obvious that these effects of the action of the thyroid drug are all well-known factors of the phenomena of Graves's disease. The following results were found in the human subject as produced by the administration of thyroid gland. These observations made by Easterbrook[23] are quoted by Edmunds: (1) loss of weight, about seven pounds a week; (2) some pyrexia; (3) some increase of perspiration; (4) diminution of the red corpuscles; (5) headache; (6) tremors; (7) an increase of pulse rate by about forty a minute; (8) an increase of the rate of respiration by about six a minute; (9) a diminution of appetite; (10) an increase of menstruation; and (11) an increased amount of urine, often albumin, but never sugar. He concludes that thyroid substance is a profound katabolic stimulant.

The present state of matters as regards thyroid therapy has been reviewed very recently by Batty Shaw,[24] who, commenting on the success of the treatment of athyrea by means of substitution products of the thyroid, remarks that nevertheless as yet no important advance has been made in the treatment of the hyperthyrea or the dysthyrea of Graves's disease. He concurs in the general view that the flooding the system with thyroidal secretion produces symptoms which are a very good imitation of the phenomena of Graves, such as tremor, nervous excitability, tachycardia, and increased metabolic activity, and that in Graves's disease the administration of thyroid may relieve the gland of any necessity for extra function. If this continues some degree of involution occurs, or the treatment may actually cause (after initial stimulation) a retrogression and atrophy. As regards the parathyroid glands, he points out that some of the nervous phenomena of Graves's disease are allied to those which result from destruction of the parathyroids. He suggests the possible utility of hypothyreic serum from thyroidectomised animals and of hyperthyreic serum from injection of thyroid extracts into animals. Collins reports one case in which Armour's parathyroid preparation was given with apparent success.

Here I have to thank Dr. Watson Williams for the opportunity of showing photographs of Mrs. B., one of the earliest cases of myxœdema treated with thyroid gland, and who has now continued treatment for over twelve years. At this time it was necessary to go to the slaughter-house and personally remove the glands when the sheep were killed, as there was no other way of obtaining them. A good result very rapidly ensued from giving two lobes minced up in a sandwich daily; but this quantity was soon found to be excessive, as it sent up the pulse to 154.

I also am able to show two photographs lent me by Mr. Edmunds of a well-known case—the one taken before the advent of the disease, and the other when the disease was well developed and before treatment commenced. Allbutt's *System*[25] gives a third portrait of this patient after treatment had restored his condition to something like its pristine one.

But now to revert again to the questions relating to the hyperthyrea of Graves's disease. It is commonly admitted that thyroidectomy produces much improvement if the patient survives the risk. The risk being, however, too great, it would be desirable that a thyrolytic serum which will serve the purpose of a partial thyroidectomy by producing a solvent action on the secreting cells, and so lead to their destruction without operative interference on the gland, should be dis covered. Many attempts to produce such a serum have been made, but so far all experimental efforts to develop such an efficient thyrolytic serum for therapeutic purposes have ended in more or less disappointment.

Lanz,[26] endeavouring to produce such a serum which will neutralise a poison formed by the cells of the patient himself, obtained good results in six cases by using the fresh milk of a thyroidless goat, the whole of the milk of one goat being consumed by the patient every twenty-four hours. When the fresh milk cannot be obtained the dried milk, sold under the name of Rodagen, may be employed instead. A remarkable case in which this was employed was reported by Dr. Murray in his Bradshaw Lecture last month.[27] Steady improvement was being made under the use of one drachm of the Rodagen thrice daily. After three weeks' treatment she became collapsed, temperature fell to 95·2°, and the heart-beat fell from 140 to 32 per minute, and continued slow for many weeks. No untoward symptom occurred later when the patient was taking smaller doses, and the patient left the hospital much improved. Dr. Murray considers that this remedy is worthy of more extended trial, but should be used with caution in doses not exceeding half a drachm. Moebius similarly employed serum prepared from the blood taken from sheep several weeks after thyroidectomy. This serum, prepared by Merck, is now to be obtained under the name of antithyroidin.[28] The serum from dethyroidised dogs and rams has also given striking results. A similar preparation, Thyroidectin, is prepared by Messrs. Parke, Davis and Co. This is a reddish-brown powder prepared from the blood of thyroidectomised animals, and sold in capsules containing five grains. One or two of these thrice daily have given encouraging results in the hands of various observers. Dr. Murray, in his recent lecture, gives results of his own attempts to prepare an anti-thyroid serum, first of all from rabbits and more recently from a goat. In neither case was he able to attribute any special effect to the use of the serum.[29]

From all these and many other endeavours it is clear that the rôle of the thyroid has of late years been amply recognised, and the various observations made gave rise to a rapid growth of opinion all over Europe that the symptom-complex is due to the production (or non-elimination) in the thyroid itself of some toxin which acts on the whole nervous system, even to the periphery, though its action mainly falls on the vaso-motor centres of the medulla and some neighbouring centres. Various papers by Arthur Maude[30] emphasised the idea that "we must regard the neurosis as a nerve poisoning for the present: the brunt undoubtedly falls on the medulla, but it is felt from the cortex to the periphery. . . . To my mind, the universal goitre, the connection with myxœdema, the results of operations on the thyroid, point to that gland as the fountain-head of evil." It is interesting to note that Dr. Michell Clarke,[31] writing on hysterical tremor, describes "a fine rapid tremor, like that present in alcoholism or in Graves's disease." Is it not possible that many cases of hysteria are due to causes similar to those which prevail in exophthalmic goitre? Bearing these facts in mind, I some years ago treated a series of cases of chorea with thyroid extract, and with very good results: the course of the disease appeared to be materially shortened.

Incomplete or rudimentary forms of the disease had been described by Trousseau, who proposed a special designation for them, which was adopted by Marie in his *Contributions a l'étude et au diagnostic des formes frustes de la Maladie de Basedow*, 1883. Trousseau had also first noticed the characteristic tremor which was more fully described by Marie, who showed it to be a constant accompanying phenomenon in the symtomatology of exophthalmic goitre. A study of these ill-developed forms of the disease, when the toxic condition probably is to be found in its milder and earlier stages, seems likely to be of much interest in the quest for an anti-toxin, and may be of great importance in relation to various functional conditions of the nervous system allied to hysteria and of the neurosal disturbances of the heart. Such a case is that of Mrs. C., now æt. 60, who has had thyroid swelling for twenty-five years or more. In 1889 twelve injections of ergotine into the gland produced some amelioration. In 1891 six injections were given, with much temporary discomfort and no permanent advantage; various other treatments

*January 12th, 1888.*

*February 4th, 1888. After Sparteine.*

*March 2nd, 1888. Also after Sparteine.*

*April, 1888.*

*October 17th, 1905. After suprarenal tabloids, gr. v., t.d.s.*

Mrs. C., æt. 60.

*Tachycardia, with very large Goitre.*

had been carried on for four years previously, beginning in 1887, for nerve and heart symptoms. In March, 1901, a friend advised Haig's American goitre cure (? thyroid), which was tried with some little relief to the throat and without any increased palpitation. In August, 1901, thyroid tablets were tried, also on the advice of a friend. Three grains twice daily after one week "reduced me, but there was no difference in the size of the gland, and it excited the heart, but relieved some of the nerve symptoms. I could not go beyond six grains daily, but could continue this for a fortnight." The patient has persistently declined all proposals to operate on the gland, and now, in 1905, the heart condition gives little trouble, but the gland is persistently causing some lateral pressure on the trachea.

Another case is that of Mrs. J., æt. 72, chronic goitre, with tachycardia of three years' duration. The heart symptoms are usually amenable to heart treatment, but the nervous tremor, malnutrition, pulsation of enlarged thyroid, venous distension in the neck, and a rapid irregular heart have continued since April, 1903. At times there has been very intractable spasmodic cough, a pseudo-bronchitis with much throat secretion, vomiting, and exhaustion threatening syncope, but sooner or later relief comes, and things go on as before. In this case the right pupil is larger than the left, and it has been observed that at times one side of the face was red and the other side white, with a sharp line of demarcation down the middle. These symptoms are at least suggestive of some grave lesion, involving the cervical portion of the sympathetic nerves, and it seems not improbable that both the heart symptoms and the goitre are of a secondary character. In both these cases suprarenal extract in five-grain tabloids has been given from time to time with some benefit. Its effect (as shown in the pulse tracing) has been to indicate a marked increase of pulse tension, although there has been little influence on the pulse rate.

Dr. Harry Campbell[32] has recently commented on the fact that Graves's disease is very apt to be overlooked, that it may remain immature for years, and that exacerbations may follow

*September 26th, 1905.* *Pulse 124. Tension low.*

*September 30th, 1905.*

*After Digitalis.*

*October 10th, 1905.* *Usual condition.*

*October 16th, 1905.* *After suprarenal tabloids*

Mrs. J., æt. 72.

*Tachycardia, with Goitre.*

fright, the climacteric, or any acute illness. We see cases of young women with tachycardia and tremor, without marked exophthalmic or pronounced goitre, although emaciation, perspiration, or pigmentation may be present. Agitation and extreme nervousness are the most striking clinical features of the disease, the entire nervous system is in a state of exaggerated irritability. The pathology centres round the thyroid gland; the passage into the blood in excessive quantity of the normal colloidal contents of the alveoli, or of a perverted thyroid secretion, leads to a rapid katabolism with increased output of carbonic acid, urea, and other excreta. Thus are explained the palpitation, emaciation, pigmentation, sweating, and the general nervousness—a condition of thyroidism, and Campbell remarks that the phenomena of the climacteric may be due to a similar cause. My patient, Mrs. C., states her experience and conviction as follows: "The complaint would not have existed but for a life of repeated and excessive nerve strain. Being caused, it would have been mended and made endurable by easier life, less care, less hard work, anxiety, and small means. Instead of other treatment, rest, bed, feeding would, I believe, have pulled me up."

The suprarenal bodies and the thymus have been also tested; but although in some isolated cases they have each been of service, it does not appear that any specific anti-toxic value can be attributed to either. As regards the preparations, it is generally admitted that suprarenal feeding, even in Addison's disease, has by no means given results comparable in brilliance with those obtained by thyroid feeding in myxœdema. An abstract of ninety-seven cases reported by Dr. E. W. Adams[33] gives results mostly disappointing. It does not, however, seem right that this drug should be summarily dismissed as useless in Graves's disease. The uses of adrenalin, suprarenin, epinephrin, paranephrin, hemisine, or by whatever name it may be called, are of such value as a local hæmostatic (and even when locally used these drugs are known to produce a general rise of blood pressure) that its stimulating effect on the heart and the increased tone of the arterial system generally should be of use in many cases of Graves's disease with heart weakness. The most familiar of these preparations is perhaps the hemisine (Burroughs, Wellcome and Co.). This the active principle of the medulla of the suprarenal capsules is in the form of a dry soluble powder. This substance must be continually passing into the blood stream, and is intimately associated with the functional integrity of the sympathetic system. Its action on the heart is to cause great acceleration of the heart beat and powerful constriction of the smaller arteries; the rise of systemic blood pressure may be so great that a secondary slowing of the heart beat is produced through the vagi; other effects are widening of the pupils and protrusion of the eyeballs. It seems clear, therefore, that this drug, like the thyroid extract itself, must only be used in Graves's disease with great caution.

As regards the action of the thymus in Graves's disease, there is a practical unanimity in the absence of any specific effect; it has been often tried and has generally been found to be practically useless. My own experience is quite in accord with this result; it has often been given, and in steadily increasing doses, and has not produced any appreciable benefit. Collins[34] states that the one patient to whom it was given showed no improvement whilst taking it. Mackenzie states that he treated twenty cases with thymus, and compared them with twenty similar cases without thymus, but could see no decided difference in the results obtained.

A case recorded by Dr. Watson Williams[35] is of especial interest, and is one of the earliest in which thymus gland was used. Symptoms were singularly unilateral, affecting the right lobe of the gland, and the right pupil was larger than the left. The condition was much aggravated by thyroid tablets; these were therefore discontinued and thymus tablets given six daily. The temperature and pulse rate both rose, and fell when the tabloids were stopped. Fresh thymus was then given, one ounce daily; in five days the pulse rose from 98 to 136, and the temperature to 99.2°. The dose being reduced to half an ounce, the pulse fell to about 110 and temperature rose in the evening. After one week the thymus was stopped. There was marked aggravation of all the symptoms, which subsided when the drug was left off, and were repeated when it was again resumed. The thymus in this case behaved in every respect as if it were thyroid.

Another case worthy of mention is the following:—

A girl, æt. 17, who developed symptoms of a severe form of Graves's disease after a fright. She had proptosis, a very large pulsating goitre, and tachycardia. She was thin, very emotional and irritable. After other methods of treatment had given no result, thymus gland, fresh from the butcher, was obtained daily, and she took six small pieces of bread spread with the finely-minced gland well sprinkled with salt. The result was decided, for she began to improve at once, and the improvement was maintained. This treatment was continued for about three months, when all the physical signs had receded, and she was able to resume her former duties. It is now ten years since the first illness; twice during that period she has developed some tachycardia with a little proptosis, but on each occasion a few weeks of treatment with thymus gland tabloids has put everything right, and she is now quite well.

The general conclusion drawn by Dr. Murray on the serum treatment is as follows:—

"We must, on the whole, conclude that at the present time in the great majority of cases the best results are obtained by general hygienic treatment combined with the use of electricity and certain drugs, and that as yet no serum or other animal product can be considered to give better results than those older methods of treatment."[36] As regards these general methods, they are considered in detail by Dr. Murray in the same lecture, and also recently by Dr. Hector Mackenzie in a lecture on the same subject.[37] He concurs with other observers that thyroid treatment generally makes the patient worse, that thymus produced in twenty cases no decided result, and that the thyroid serum of Moebius gave no good effects, and that as yet we have only to trust to the older methods of treatment on the lines indicated; with those, however, "with care and perseverance there is a fair chance of our efforts being rewarded by the recovery of the patient."

Dr. Kenneth Wills tells me that he has of late obtained some fairly good results from X ray and other electrical treatment of this disease.

One question remains on which it is essential that something further must be said. It has frequently been stated that operative treatment gives good evidence in favour of the thyroid origin of the disease; doubtless this is so, but at what cost is this knowledge gained? The mortality is well known to be exceedingly high, and in spite of the apparently favourable reports of Kocher and others, it is generally admitted that the results are, in Osler's words, "notoriously uncertain." In these days only partial removal would ever be advocated, and this usually as advised by Kocher with the help of a local anæsthetic, never with ether or chloroform, and bearing in mind that thyroidectomy is much more fatal to the young than to adults. Murray remarks that the risks of surgical treatment are so great that he does not consider partial thyroidectomy is advisable; in two cases in which he had seen this operation performed both patients died within an hour or two. Mackenzie also states that two of his patients had died as a direct result of the operation. I can recall other cases in which death took place within a few hours.

One of these was that from which some of my photographs were prepared,[38] that of a woman, æt. 34, admitted on June 17th, 1895, with the ordinary symptoms of Graves's disease, of medium intensity. After six weeks with little indication of improvement it was decided to operate. A surgical colleague advised this, and at the end of July removed the right lateral lobe. The result was disastrous, though the operation was quickly done, and the bleeding was slight; yet in about thirty hours the patient died with pyrexia, rapid pulse, and delirium, culminating in heart failure.

In a case recorded by Dr. Arthur J. Hall[39] two operations were successfully performed, but with only partial and very temporary improvement, although the whole of the right lobe and the isthmus had been removed. I am indebted to Dr. Hall for the further report of the case, from which it appears that a third operation—the removal of the rest of the thyroid—was speedily followed by tetany, bronchitis, and death.

Collins[40] states that his experience with surgical treatment has been most unfortunate—of three cases, all died immediately.

Another of my Infirmary cases gave us a very satisfactory result. Æt. 25, admitted March 12th, 1895, with thyroid swelling of nine months' duration, followed by proptosis, wasting, amenorrhœa, cardiac symptoms, and wasting. Both lobes of the thyroid were equally enlarged, the isthmus also. Bruits and tremor of the usual character existed, with a pulse of 120. On March 20th Mr. Harsant removed the right lobe and a portion of the isthmus. No bad symptoms followed, and on March 20th the pulse had come down to 90, and there was less tremor. April 27th: She had gained 12 lbs. in weight, general health had much improved, and she was discharged fairly well.

Further, it has been found that thyroidectomy, even when successfully and safely performed, has not always been successful in curing the disease; in some cases only partial improvement has occurred, too much gland having been left behind. Various other operations have been from time to time performed, such as division of the isthmus and ligature of the thyroid arteries; these may be less dangerous, but are also less likely to be of any lasting service.

Dreyfus[41] gives much more favourable results from operative treatment. Complete recovery followed operation in 75·7 per cent., great improvement in 10 per cent., slight in 2·4, and death in 7·9 per cent. When it is remembered that only severe cases were operated on the author believes that these results are better than can be reported from any other method of treatment.

Kocher's well-known results are also in favour of operative interference.

Accordingly, on the theory that the sympathetic system may be the *fons et origo mali*, it has been thought that operations with a view to the excision of more or less of the cervical ganglia and their connecting nerves may be more successful than have been those on the thyroid. Edmunds[42] gives an analysis by Boisson of twenty-seven cases: in seven of these no improvement followed; the last case of all was one in which the patient lost her eyesight, and finally died from severe Graves's disease ten weeks after the sympathetic operation on one side, and seven weeks after its removal on the other side, thus showing that the sympathetic was not the cause of her symptoms. Edmunds also quotes Berry, who at the Royal Medical and Chirurgical Society in the previous year expressed the opinion that the sympathetic has nothing whatever to do with Graves's disease, and that the sympathetic operation should never be performed. Farquhar Curtis, of New York,[43] reports that out of seven cases operated on by sympathectomy two died from acute thyroidism, one relapsed nine months after operation, and died from the original disease with acute endocarditis, and only one was completely cured five years after operation. He preferred the operation of thyroidectomy, as it could be easier done under a local anæsthetic, and the mortality was not so high.

The almost universal acceptance of the thyroid as the primal cause of the group of symptoms first described by Graves makes it all the more disappointing that, although on this theory some anti-toxin is not unlikely to be found which will neutralise the action of the enlarged and over-active gland, and in spite of a vast deal of patient investigation, we are driven to the conclusion that the problem has not yet been solved. It may be that although pathologists may be on the right track, yet some complicating agency must be thwarting their endeavours, and these opposing influences are not unlikely to be found in the nerve centres.

One thing is clear, that general hygienic treatment and that directed to the condition of nerves and heart still remains paramount. It may be that we are only dealing with symptoms, but these can be dealt with more or less efficiently on the lines which have long existed. The neuro-cardiac theory is then the foundation on which our treatment must still be based, and although we have as yet no specific rigid line to follow, yet the recognition of the phenomena of Graves's disease, in any given case of cardiac and nerve symptoms, gives us encouragement to expect that the symptoms will be more amenable to treatment than are other kinds of serious disturbances of the heart and nerves. So far as these structures are concerned we may consider the phenomena of the milder forms of exophthalmic goitre to be in their essence functional, and as such curable; their continued study is likely to give us material assistance and encouragement in dealing with other toxic conditions of the nervous centres and cardiac nerves.

And here, gentlemen, I conclude my imperfect sketch of the present views of this interesting malady: the toxic questions are new since Fox wrote on the subject, and the anti-toxic questions are by no means yet worked out. There are many problems still awaiting solution by those who have time and ability for the accomplishment. Fox still speaks to us, and his message is, "Whatsoever thy hand findeth to do, do it with thy might; for there is no work, nor device, nor knowledge, nor wisdom, in the grave whither thou goest."

N.B.—I have to express my thanks to Mr. Walter Edmunds for many of the illustrations in the text, also to him and to Sir Victor Horsley, Dr. George Murray, and Dr. Edgeworth for lantern slides with which the lecture was illustrated. My thanks are also due to Mr. James Taylor and to Dr. Imlay for much photographic assistance.

### BIBLIOGRAPHY

1. **Edward Long Fox**—The Influence of the Sympathetic on Disease. London, 1885.
2. **Greenfield**—*Brit. M. J.*, 1893, ii. 1266.
3. **Tedeschi**—Gould's *Year Book*, 1904, p. 405.
4. **Shingleton Smith**—*M. Times and Gaz.*, 1878, i. 647; *Tr. Internat. M. Cong.* (London), 1881, ii. 80.
5. **Graves**—Clinical Medicine, vol. ii., 1884, p. 220 (New Syd. Soc.).
6. **Horsley**—*Brit. M. J.*, 1896, ii. 1623.
7. **Michell Clarke**—*Bristol M.-Chir. J.*, 1887, v. 17.
8. **Horsley**—*Lancet*, 1886, ii. 1163.
9. „ —*Brit. M. J.*, 1896, ii. 1624.
10. **Edmunds**—*J. Path. and Bacteriol.*, 1898, v. 33.
11. **Murray**—*Lancet*, 1899, i. 747.
12. **Hutchison**—*Brit. M. J.*, 1897, i. 194.
13. **Edmunds**—*Tr. Path. Soc. Lond.*, 1896, xlvii. 235.
14. **MacCallum and Davidson**—*Med. News*, 1903, lxxxiii. 820; 1905, lxxxvi. 625.
15. **Gley**—*Brit. M. J.*, 1901, ii. 771.
16. **Walsh**—*Am. Med.*, 1905, ix. 815.
17. **Humphry**—*Lancet*, 1905, ii. 1390.

18. **Mackenzie**—*Brit. M. J.*, 1905, ii. 1077.
19. **Sajous**—The Internal Secretions and the Practice of Medicine, vol. i., 1903.
20. **Maurice Faure**—quoted by Sajous, *op. cit.*, p. 162.
21. **Osler**—Æquanimitas (Lewis, London), p. 271.
22. **Hutchison**—*Brit. M. J.*, 1898, ii. 142.
23. **Easterbrook**—*Lancet*, 1898, ii. 546.
24. **Batty Shaw**—Organotherapy, 1905.
25. **Edmunds**—Allbutt's System of Medicine, vol. iv., 1897.
26. **Lanz**—*München. med. Wchnschr.*, 1903, l. 146.
27. **Murray**—*Brit. M. J.*, 1905, ii. 1250.
28. **Hempel and Thienger**—*München. med. Wchnschr.*, 1905, lii. 14, 15.
29. **Murray**—*loc. cit.*, p. 1251.
30. **Maude**—*Tr. M. Soc. Lond.*, 1894, xvii. 12; *Brain*, 1894, xvii. 246.
31. **Michell Clarke**—Hysteria and Neurasthenia, 1905.
32. **Campbell**—*Brit. M. J.*, 1902, ii. 1420.
33. **Adams**—*Practitioner*, 1903, lxxi. 472.
34. **Collins**—*Post-Graduate*, 1905, xx. 470.
35. **Watson Williams**—*Clin. J.*, 1895–96, vii. 93.
36. **Murray**—*loc. cit.*
37. **Hector Mackenzie**—*loc. cit.*
38. **Edgeworth**—*Bristol M.-Chir. J.*, 1896, xiv. 41.
39. **Hall**—*Clin. J.*, 1895–96, vii. 38.
40. **Collins**—*loc. cit.*
41. *Brit. M. J.*, 1905, ii. Epitome, 87.
42. **Edmunds**—Erasmus Wilson Lectures—*Lancet*, 1901, i. 1317, 1381, 1449.
43. **Curtis**—*Med. Rec.*, 1905, lxviii. 715.

## Figures and Tables

**Figure f1:**
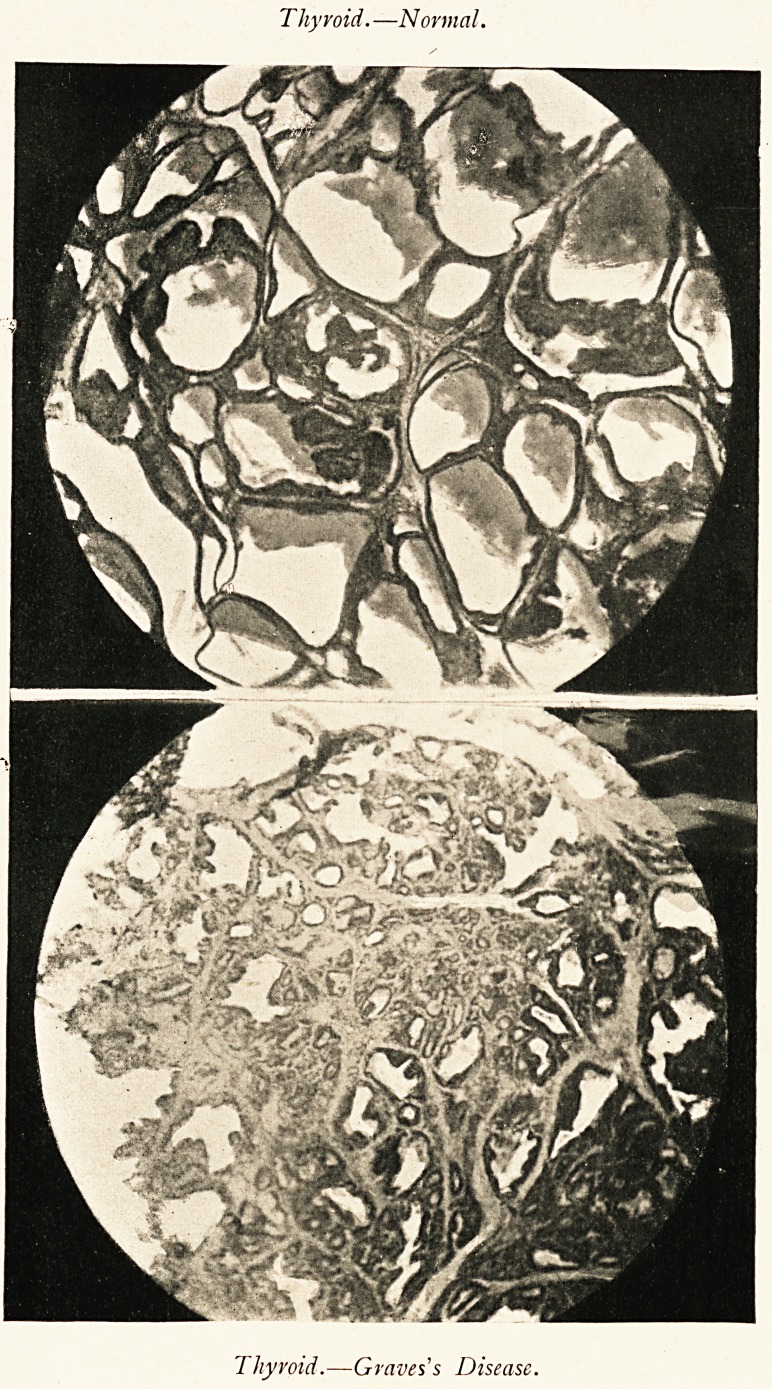


**Fig. 1. f2:**
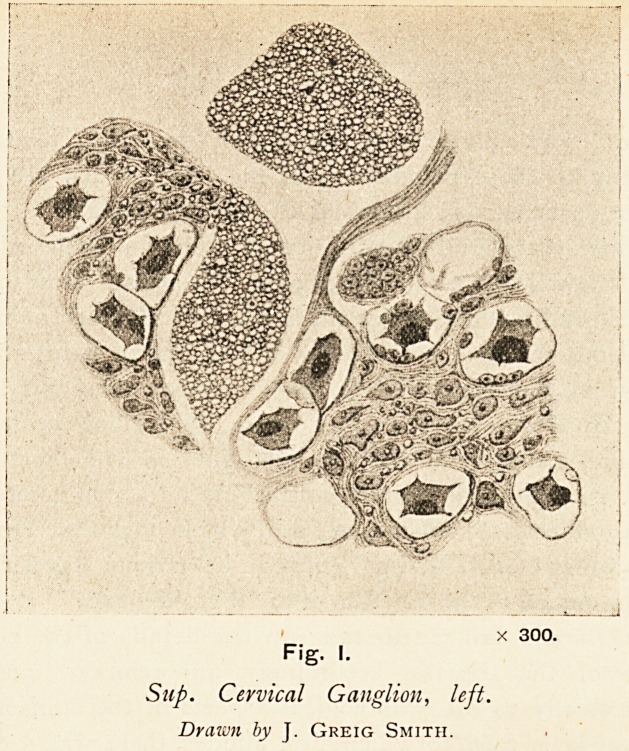


**Fig. 2. f3:**
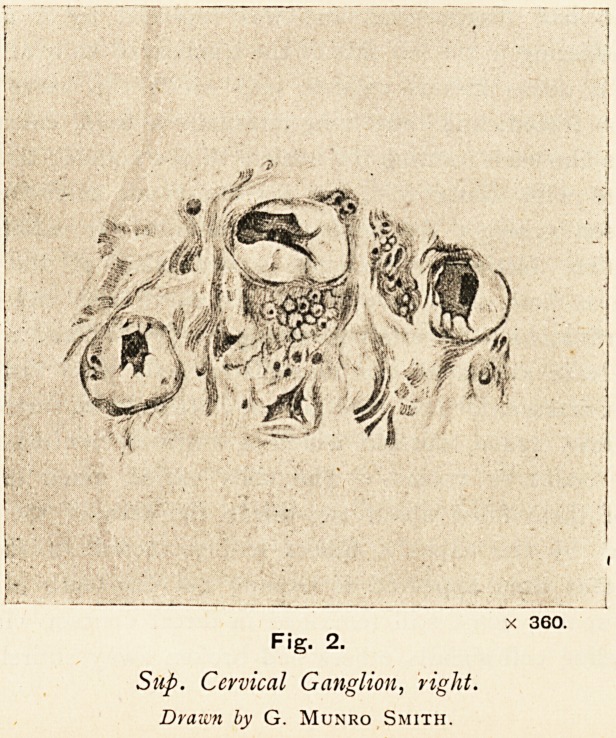


**Fig. 3. f4:**
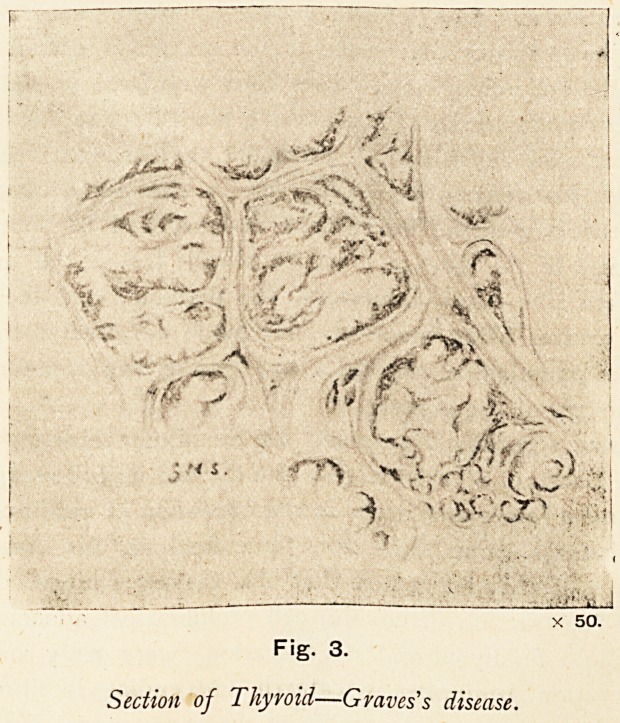


**Fig. 4. f5:**
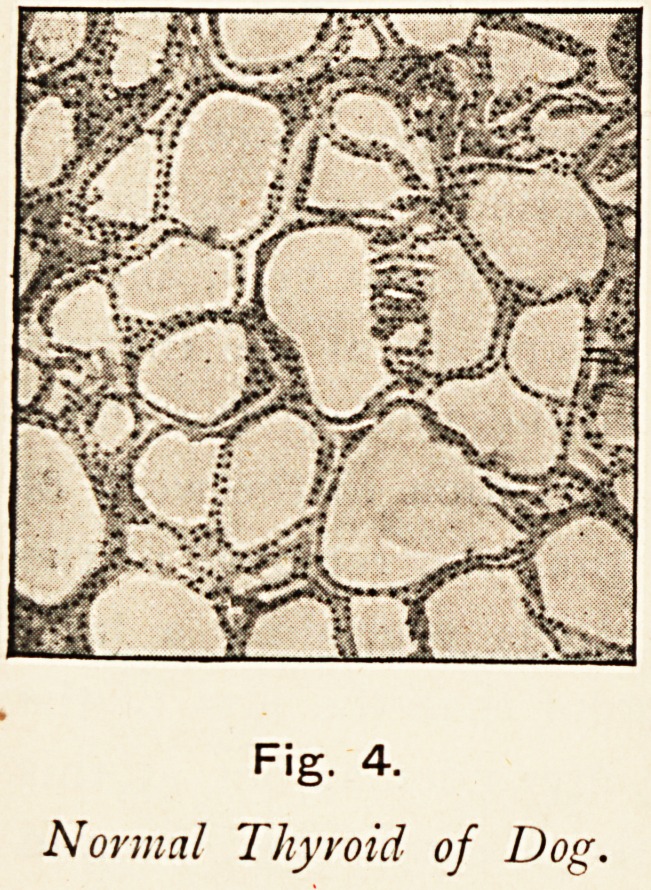


**Fig. 5. f6:**
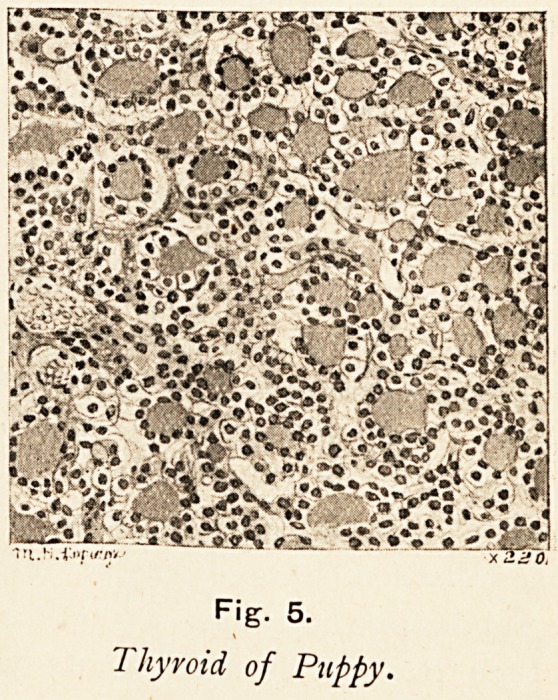


**Fig. 6. f7:**
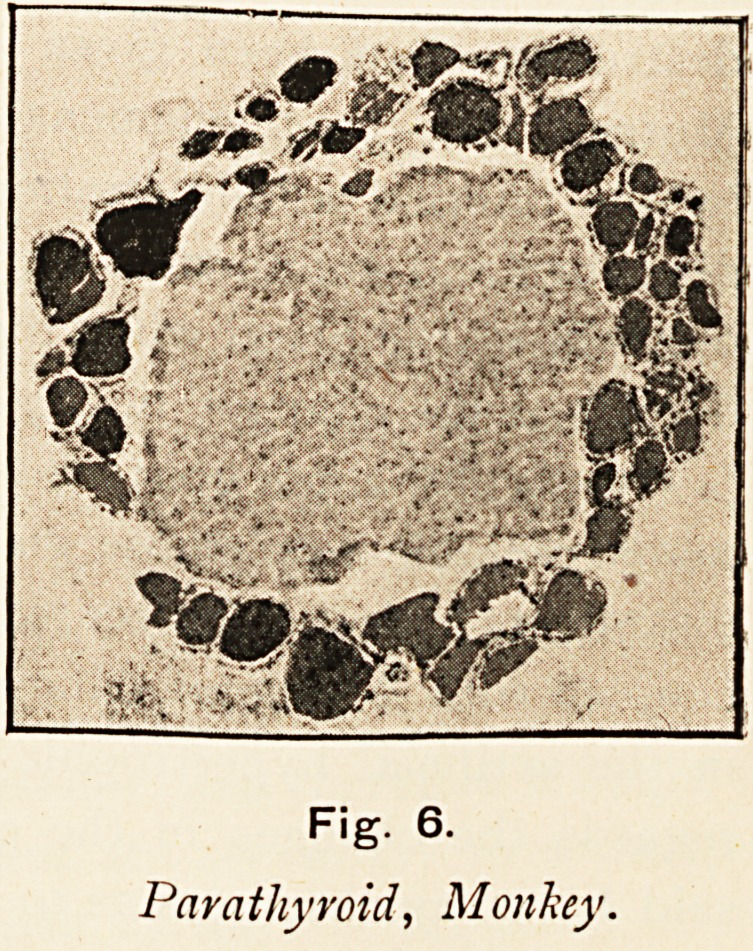


**Fig. 7. f8:**
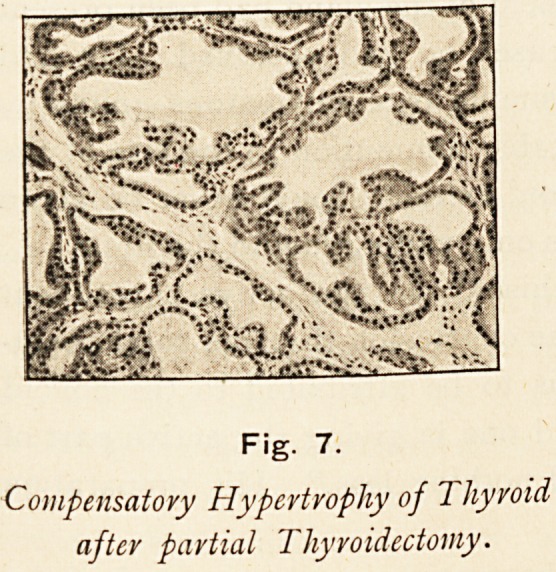


**Fig. 8. f9:**
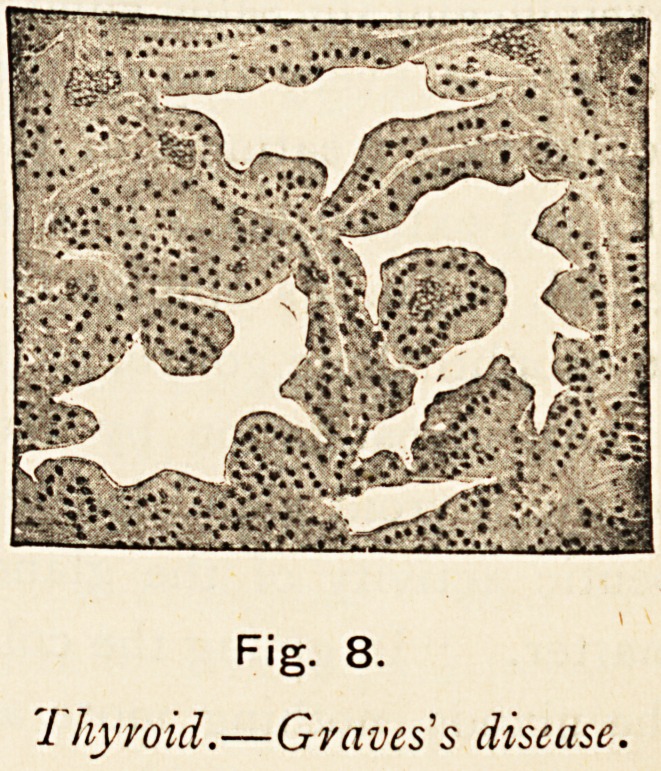


**Fig. 9. f10:**
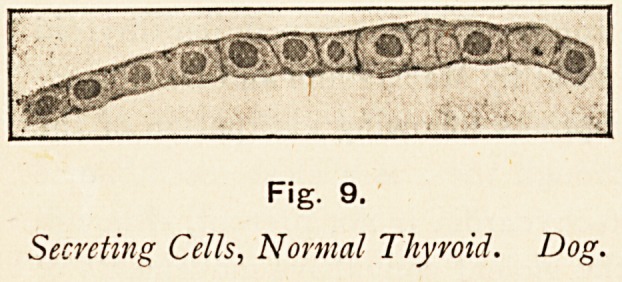


**Fig. 10. f11:**
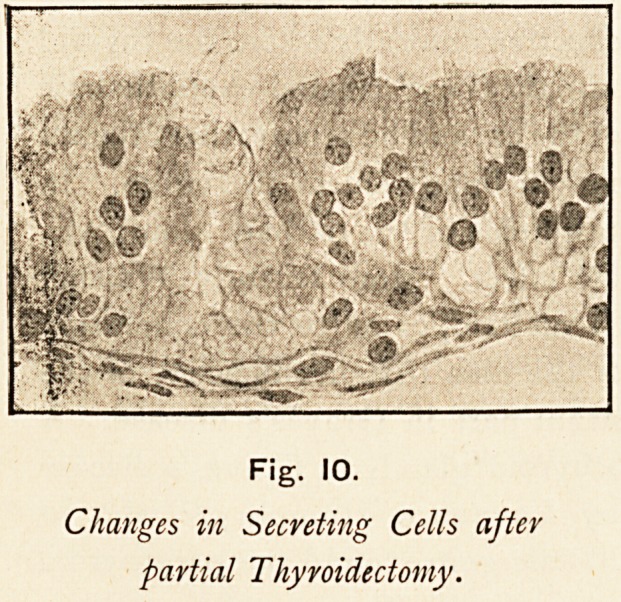


**Fig. 11. f12:**
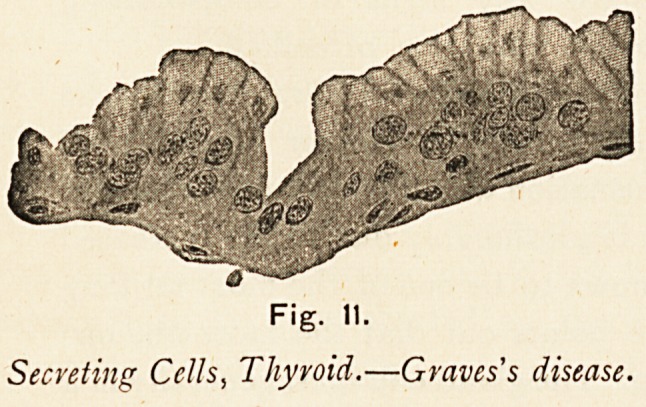


**Figure f13:**
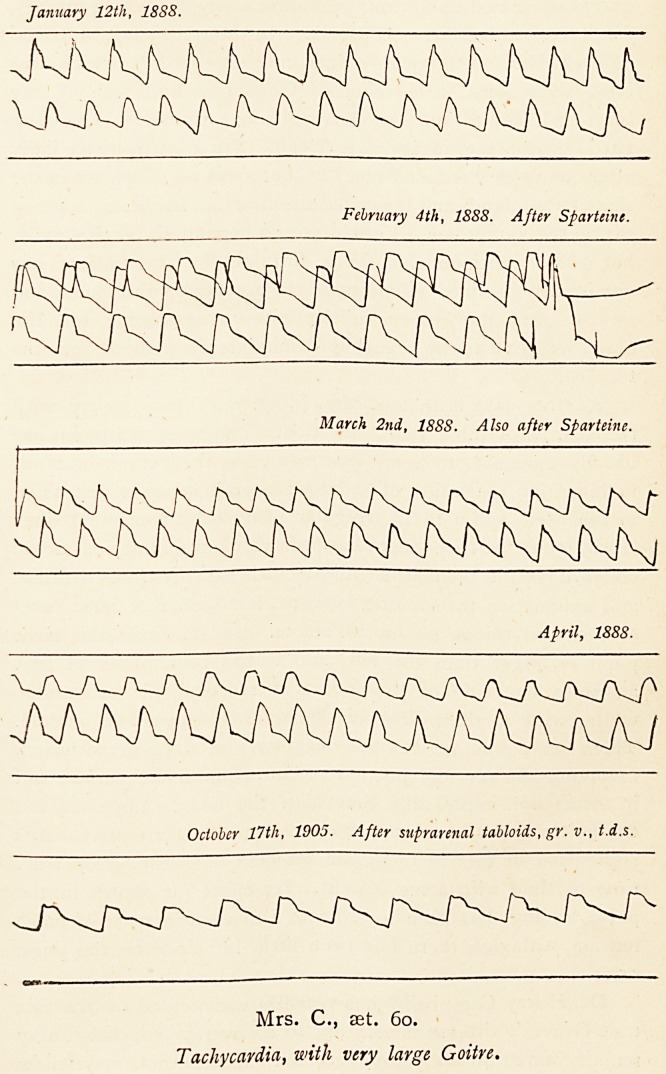


**Figure f14:**